# Increased urinary prostaglandin E2 metabolite: A potential therapeutic target of Gitelman syndrome

**DOI:** 10.1371/journal.pone.0180811

**Published:** 2017-07-10

**Authors:** Xiaoyan Peng, Lanping Jiang, Chen Chen, Yan Qin, Tao Yuan, Ou Wang, Xiaoping Xing, Xuemei Li, Min Nie, Limeng Chen

**Affiliations:** 1 Department of Nephrology, Peking Union Medical College Hospital, Chinese Academy of Medical Sciences & Peking Union Medical College, Beijing, China; 2 State Key Laboratory of Medical Genetics, Department of Pediatrics, Xiangya Hospital, Central South University, Changsha, China; 3 Department of Endocrinology & Key Laboratory of Endocrinology, National Health and Family Planning Commission, Peking Union Medical College Hospital, Chinese Academy of Medical Sciences & Peking Union Medical College, Beijing, China; University of Utah School of Medicine, UNITED STATES

## Abstract

**Background:**

Gitelman syndrome (GS), an inherited autosomal recessive salt-losing renal tubulopathy caused by mutations in *SLC12A3* gene, has been associated with normal prostaglandin E2 (PGE2) levels since 1995 by a study involving 11 clinically diagnosed patients. However, it is difficult to explain why cyclooxygenase-2 (COX2) inhibitors, which pharmacologically reduce PGE2 synthesis, are helpful to patients with GS, and few studies performed in the last 20 years have measured PGE2 levels. The relationships between the clinical manifestations and PGE2 levels were never thoroughly analyzed.

**Methods:**

This study involved 39 GS patients diagnosed by *SLC12A3* gene sequencing. Plasma and 24-h urine samples as well as the clinical data were collected at admission. PGE2 and PGEM levels were detected in plasma and urine samples by enzyme immunoassays. The in vivo function of the sodium-chloride co-transporter (NCC) in GS patients was evaluated using a modified thiazide test. The association among PGE2 levels, clinical manifestations and the function of NCC in GS patients were analyzed.

**Results:**

Significantly higher levels of urinary and plasma PGEM were observed in GS patients than in the healthy volunteers. Higher urinary PGEM levels indicated more severe clinical manifestations and NCC dysfunction estimated by the increase of Cl^-^ clearance. A higher PGEM level was found in male GS patients, who showed earlier onset age and more severe hypokalemia, hypochloremia and metabolic alkalosis than female GS patients. No relationship between renin angiotensin aldosterone system activation and PGEM level was observed.

**Conclusions:**

Higher urinary PGEM levels indicated more severe clinical manifestations and NCC dysfunction in GS patients. COX2 inhibition might be a potential therapeutic target in GS patients with elevated PGEM levels.

## Introduction

Gitelman syndrome (GS, OMIM 263800) is an inherited autosomal recessive salt-losing renal tubulopathy. It is mainly caused by loss-of-function mutations in the *SLC12A3* gene encoding the sodium-chloride co-transporter (NCC) in the distal convoluted tubule (DCT)[1]. Patients with GS are treated by oral potassium and magnesium supplementation and potassium-sparing diuretics. Due to its inhibition of prostaglandin E2 (PGE2) synthesis in the kidney, indomethacin was traditionally used in patients affected by Bartter syndrome (BS)[2], another salt-losing tubulopathyies caused by mutations in genes coding for proteins responsible for salt reabsorption in the loop of Henle. It has long been believed that NCC disorders are not associated with markedly elevated renal PGE2 synthesis, especially in adult patients [3]. Studies in several case series indicated that indomethacin, a nonselective inhibitor of cyclooxygenase (COX), can improve hypokalemia and developmental delays [2, 4–11]. Recently, an open-label, randomized, crossover study confirmed the efficiency of indomethacin treatment in GS patients via a significant decrease in renin activity and the estimated glomerular filtration rate (eGFR)[12]. However, no reliable laboratory measurements support indomethacin therapy. Direct evidence of plasma and urinary PGE2 levels is still limited to that reported in the study by Luthy et al. from 11 GS patients in 1995[3].

In vivo, PGE2 is rapidly converted to its 13,14-dihydro-15-keto metabolite, with more than 90% of circulating PGE2 cleared by a single passage through the lungs[13]. This metabolite is chemically unstable and undergoes a variable amount of degradation to prostaglandin A (PGA) products. Thus, plasma and urine samples from patients actually contain very little intact PGE2, and the PGE2 metabolites (PGEM) can be measured[14] to provide a more reliable estimate of actual PGE2 production.

In this study, we measured the levels of PGE2 and PGEM in plasma and urine in genetically diagnosed GS patients. The associations between PGE2 and clinical characteristics were analyzed, and the NCC function was also evaluated by the modified thiazide test[15].

## Materials and methods

The study was approved by the Ethics Committee on Human Studies at Peking Union Medical College Hospital (PUMCH), Chinese Academy of Medical Sciences, Beijing, China. The authors adhered to the Declaration of Helsinki, and patients were included after providing informed consent.

### Subjects

From April 1, 2013 to April 1, 2016, patients at PUMCH with clinically suspected GS were recruited to the study. The diagnostic evidence included persistent hypokalemia excluding medicinal or gastrointestinal causes, metabolic alkalosis, normotension or hypotension, with or without hypomagnesemia and hypocalciuria. The components of the renin-angiotensin system were always activated [16]. Age-matched healthy volunteers were recruited as controls in October 1, 2015 to April 1, 2016. Authors had access to information that could identify individual participants after data collection.

### *SLC12A3* gene sequencing

The *SLC12A3* gene encoding the NCC was sequenced directly as we previously described [17]. Briefly, genomic DNA was isolated and purified from peripheral blood lymphocyte of the patients and used for polymerase chain reaction amplfication of individual exons of the *SLC12A3* gene. A total of 23 pairs of oligo-nucleotide primers were generated to amplify all 26 exons and flanking intronic regions (to detect the mutations in intron-exon boundaries of the conventional 5’ GT and 3’ AG consensus splice sites) of the *SLC12A3* gene. Sanger direct sequencing was performed on an ABI3730xl automated DNA sequencer (Life Technologies Division Applied Biosystems, The Netherlands) by BGI (Beijing, China). GenBank accession number NM_000339.2 was used as a reference sequence, in which the A of ATG was number 1.

### Clinical data

The individual clinical manifestations were documented; serum and urinary biochemical parameters, arterial blood gas analysis and electrocardiogram (ECG) examinations were monitored via routine clinical evaluations. The eGFR was calculated by the Chronic Kidney Disease Epidemiology Collaboration equation (CKD-EPI). Hypomagnesemia was defined as any recorded serum total magnesium level <0.7 mmol/L (serum magnesium reference: 0.70–1.10 mmol/L). Blood was collected from patients in the upright position and was used to test the plasma renin activity (PRA) and the angiotensin II (AngII) and aldosterone levels by radioimmunoassay.

### Thiazide test

Patients underwent the thiazide test according to the protocol described in our previous studies[15, 18]. Briefly, after an overnight fast, the subjects were asked to drink 10 mL/kg body weight of water in 15 min immediately before the test. Two continuous 30-min urine samples were collected as the “basal” clearance. Then, 50 mg of hydrochlorothiazide (HCT) was administered orally, and 6 additional 30-min clearance tests were performed. Blood samples were obtained at 60 and 240 min. The clearance rates (CRs) of sodium and chloride were evaluated.

### Specimen collection

Twenty-four-hour urine specimens were collected from the first morning (7 am) to the second morning (7 am). Plasma specimens (4 mL) were collected using an anticoagulant EDTA-K2 vacuum blood collection tube with 10 μL (10 mmol/L) indomethacin (TGI, Japan) preprocessed. Samples were promptly transported to the laboratory after collection. Each sample was aliquoted into 2 mL cryovials and stored at −80°C within 60 min of being obtained from the study participants. All samples were stored at −80°C until analysis.

### PGE2 and PGEM measurement

The urinary PGE2 concentration was determined using a PGE2 expression kit (PGE2 EIA Kit, Cayman Chemical Co., Ann Arbor, MI). A PGEM assay kit (CAYMAN 514531, Cayman Chemical Co., Ann Arbor, MI) was used to measure urinary and plasma PGEM levels. The assay was performed according to the manufacturer’s instructions with appropriate dilutions.

### Statistical methods

Normally distributed variables are expressed as the means±SD and compared using the independent samples T test. Non-normally distributed parameters are expressed as medians (25^th^, 75^th^ percentiles), and non-parametric tests were used to compare the differences between two groups. The paired t-test was used to compare individual fractional excretions of electrolytes before and after HCT application. The association between urinary PGEM and plasma electrolytes was evaluated by Spearman’s correlation analysis. Differences were considered significant when *P*<0.05. All statistical analyses were performed with the SPSS statistical software 14.0 (SPSS, Chicago, IL).

## Results

### 1. General information of GS patients

26 male and 13 female patients with a median age of 26 years were genetically diagnosed with GS (S1 Table). Among them, 5, 22, and 12 were homozygous, compound heterozygous and heterozygous, respectively. A total of 44 *SLC12A3* gene mutations was detected, including 35 missense mutations, 3 frame shift mutations, 3 nonsense mutations, 2 splice site mutations and 1 insertion mutation. Muscle weakness (76.9%) and fatigue (56.4%) were most frequently reported by patients. The main clinical and biochemical profiles of GS patients are listed in Table 1. Compared to female patients, male GS patients showed earlier median onset age (19.0 years vs. 31.0 years) and more severe hypokalemia (3.48±0.53 mmol/L vs. 3.99±0.55 mmol/L, *P<*0.05), hypochloremia (95.6±3.0 vs. 99.1±3.3 mmol/L, *P<*0.05) and metabolic alkalosis (cHCO_3_^-^: 30.2±3.5 vs. 26.4±3.6 mmol/L, *P<*0.01; median ABE: 5.7 vs. 3.1 mmol/L, *P*<0.05).

**Table 1:** Clinical and biochemical profile of GS patients.

Items	Total GS (n = 39)	Male(n = 26)	Female(n = 13)
**General information**			
Age(years)	26.0(19.0, 42.0)	24.5(16.8, 41.0)	31.0(22.0, 42.0)
Onset age(years)*	22.0(12.0, 31.0)	19.0(12.0, 27.0)	31.0(15.5, 40.5)
BMI(kg/m^2^)	21.51±3.69	22.19±3.72	19.27±2.69
eGFR	119.8±22.0	117.0±24.2	125.6±15.8
SBP(mmHg)*	109.1±12.0	112.2±9.8	103.0±4.0
DBP(mmHg)	71.8±9.7	73.3±8.9	69.0±10.9
QTc(sec)	0.432(0.416, 0.455)	0.432(0.413, 0.449)	0.454(0.418, 0.467)
**Serum(mmol/L)**			
Potassium*	3.15±0.59	2.99±0.55	3.48±0.53
Natrium	138.4±2.1	138.7±2.3	137.8±1.5
Chloride*	96.8±3.5	95.6±3.0	99.1±3.3
Magnesium	0.691±0.171	0.664±0.180	0.744±0.143
Calcium	2.430±0.134	2.439±0.142	2.412±0.120
Phosphate	1.219±0.298	1.206±0.342	1.245±0.188
**24h Urine(mmol/d)**			
Potassium	101.6±71.8	90.0±41.8	126.9±111.2
Natrium	226.0±84.4	232.7±83.5	211.6±88.4
Chloride	259.6±76.1	257.7±77.4	264.0±76.4
Magnesium	4.46(3.91, 6.69)	4.69(3.93, 7.69)	4.28(3.79, 5.22)
Calcium	1.219(0.620, 2.739)	1.168(0.650, 2.550)	1.580(0.370, 2.765)
Phosphate	14.85(11.00, 24.17)	16.64(10.45, 24.83)	13.86(10.83, 20.65)
Ca/Creatinine*	0.058(0.029, 0.112)	0.055(0.028, 0.111)	0.062(0.048, 0.271)
**Arterial blood gas**			
pH	7.469±0.038	7.464±0.032	7.478±0.047
cHCO_3_^-^(mmol/L)**	28.90±3.95	30.18±3.52	26.35±3.63
ABE(mmol/L)*	4.86±2.94	5.73±2.61	3.11±2.86
**Renin–Angiotensin-Aldosterone System**			
Renin(ng/ml/h)	2.610(1.265, 6.608)	3.065(1.265, 6.608)	2.375(1.233, 9.660)
AngII(pg/ml)	238.48(153.15, 377.11)	213.21(156.56, 458.00)	265.75(95.30, 324.17)
Ald(ng/dl)	17.58(14.03, 25.85)	17.15(14.05, 23.65)	20.86(13.86, 26.15)

Abbreviations: ABE, actual base excess; DBP, diastolic blood pressure; eGFR, estimated glomerular filtration rate, ml/min/1.73m^2^; GS, Gitelman syndrome; SBP, systolic blood pressure; Normal range of Renin: 0.93–6.56ng/ml/h, AngII: 25.3–143.3pg/ml, Ald: 6.5–29.6 ng/dl.

*P<0.05

**P<0.01 when compared between male GS patients and female GS patients.

### 2. Higher PGEM levels in GS patients

**Fig 1:**
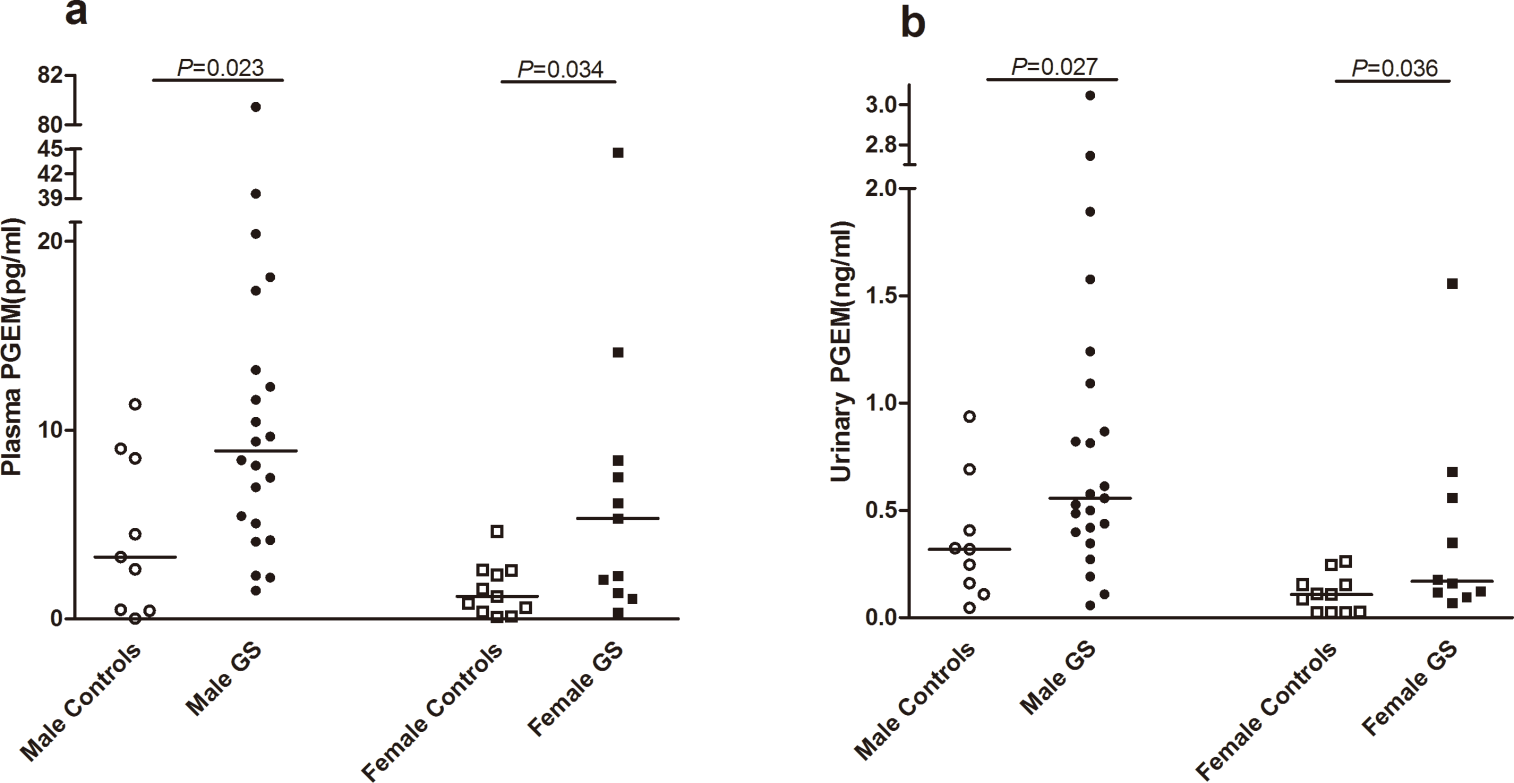
The median PGEM level in the plasma and urine samples of Gitelman syndrome patients. **(a)** The median plasma PGEM level of GS patients and healthy controls. GS patients showed a higher median plasma PGEM level than healthy controls of the same gender. (**b)** The PGEM concentration of 24-h urine was higher in GS patients. Both male and female GS patients had a higher PGEM level than controls. The median concentration of plasma and urinary PGEM in male individuals was higher than that of females, both in GS patients and controls. Abbreviations: GS, Gitelman syndrome; PGEM, prostaglandin E2 metabolite.

The PGEM levels of plasma and 24-h urine samples from males were higher than those of females with or without GS. As shown in Fig 1, the median plasma PGEM level of male GS patients was approximately 2.7 times that of healthy male controls (8.90 vs. 3.27 pg/mL, *P* = 0.023). In females, the gap increased to 4.5 times between GS patients and healthy controls (5.327 vs. 1.193 pg/mL, *P* = 0.034). The 24-h urinary PGEM concentrations in male and female GS patients were much higher than in healthy controls of the same gender. No significant differences were observed in 24-h urine PGE2 concentrations between patients and healthy controls.

### 3. Association of PGEM with clinical characteristics of GS patients

**Fig 2:**
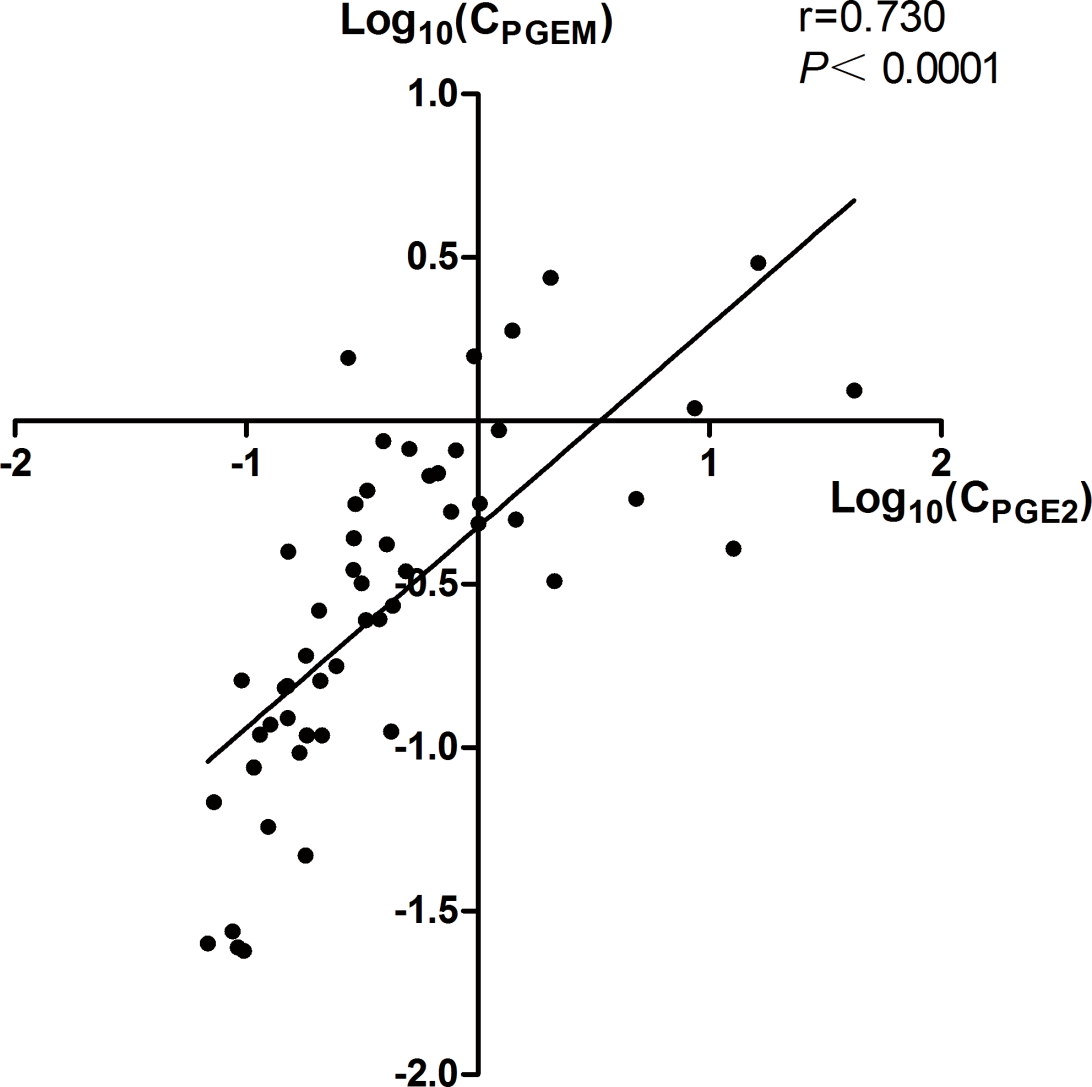
Individual urinary prostaglandin E2 (PGE2) and PGE metabolite (PGEM) levels (33 GS patients and 20 healthy controls; log transformed). Urinary PGEM and PGE2 levels showed a good correlation (r = 0.730, *P*<0.001). Abbreviations: C_PGEM_, 24h urinary prostaglandin E2 metabolite concentration; C_PGE2_, 24h urinary prostaglandin E2 concentration.

The urinary PGE2 concentration varied in each individual, ranging from 0.07 ng/mL to 42.02 ng/mL, while the PGEM concentration ranged from 0.02 ng/mL to 3.04 ng/mL. The individual urinary PGEM levels exhibited a good correlation with the urinary PGE-2 level (r = 0.707, *P*<0.001, Fig 2), but were not associated with the plasma PGEM level (n = 27, r = 0.358, *P* = 0.066). Table 2 showed that the urinary PGEM concentration was associated with serum chloride (r = -0.409, *P* = 0.018) and magnesium levels (r = -0.374, *P* = 0.032), urinary calcium excretion (r = -0.443, *P* = 0.013) and arterial HCO3^-^ concentration (r = 0.428, *P* = 0.013). Serum calcium (r = 0.553, *P*<0.001) levels and the urinary Ca/Cr ratio (r = -0.369, *P* = 0.045) were also associated with plasma PGEM.

**Table 2:** Linear correlation analyze among prostaglandin E2 metabolite concentration and clinical parameters.

	Plasma-PGEM (pg/ml)		Urine-PGEM (ng/ml)	
	r	*P*	r	*P*
Age (years)	-0.200	0.263	0.009	0.961
Onset age (years)	-0.290	0.101	-0.075	0.678
BMI(kg/m^2^)	0.143	0.505	0.093	0.657
SBP(mmHg)	0.164	0.396	0.138	0.476
eGFR	0.068	0.715	-0.019	0.918
QTc(sec)	-0.048	0.826	-0.116	0.580
Serum K^+^(mmol/L)	-0.146	0.418	-0.200	0.266
Serum Na^+^(mmol/L)	0.091	0.615	0.269	0.130
Serum Cl^-^(mmol/L)	-0.286	0.107	-0.409	**0.018**
Serum Mg^2+^(mmol/L)	0.032	0.860	-0.374	**0.032**
Serum Ca^2+^(mmol/L)	0.553	**0.001**	-0.059	0.746
Serum P(mmol/L)	0.086	0.632	-0.084	0.640
24h Urinary K^+^(mmol/d)	0.117	0.518	0.014	0.938
24h Urinary Na^+^(mmol/d)	0.061	0.735	0.349	**0.050**
24h Urinary Cl^-^(mmol/d)	0.006	0.972	0.300	0.102
24h Urinary Mg^2+^(mmol/d)	0.219	0.238	0.094	0.615
24h Urinary Ca^2+^(mmol/d)	-0.091	0.621	-0.443	**0.013**
24h Urinary P(mmol/d)	0.097	0.605	-0.035	0.853
Urinary Ca^2+^/Cr ratio	-0.369	**0.045**	-0.108	0.585
Blood gas cHCO_3_^-^(mmol/L)	0.098	0.589	0.428	**0.013**
Blood gas ABE(mmol/L)	0.119	0.508	0.544	**0.001**
Upright Renin(ng/ml/h)	-0.040	0.838	0.171	0.376
Upright Angiotensin II(pg/ml)	0.325	0.085	0.073	0.707
Upright Aldosterone(ng/dl)	-0.138	0.482	0.004	0.982
ΔClearance Cl in HCT test(ml/min)	0.130	0.619	-0.321	0.226

Abbreviations: ABE, actual base excess; eGFR, estimated glomerular filtration rate, ml/min/1.73m^2^; GS, Gitelman syndrome; SBP, systolic blood pressure; PGEM, prostaglandin E2 metabolite; ΔClearance Cl, the absolute increases in the chloride learance rate; HCT, hydrochlorothiazide

### 4. Higher urinary PGEM levels predict more severe NCC dysfunction

GS patients were divided into two groups by the cut-off point of the 50% cumulative frequency of the median urinary PGEM concentration (males 0.557 ng/mL, females 0.160 ng/mL). Compared to patients in the lower PGEM group(Table 3), patients in the higher PGEM group showed higher percentages of nocturia (66.7% vs. 26.7%, *P* = 0.037) and dyspnea (27.8% vs. 0, *P* = 0.049) and more severe metabolic alkalosis (higher ABE, *P* = 0.033) with higher daily urinary excretion of potassium (272.8±89.8 mmol/d vs. 179.0±55.7 mmol/d, *P* = 0.002) and chloride (304.1±83.6 mmol/d vs. 211.2±39.8 mmol/d, *P<*0.001). No significant differences in onset age, blood pressure and kidney function were shown between the two groups.

**Table 3:** Clinical data of patients with lower and higher urinary PGEM levels.

Items	Lower Urinary PGEM (N = 15)	Higher Urinary PGEM (N = 18)	*P* value
General information			
Male (n, %)	11, 73.0%	12, 66.7%	0.722
Age(years)	26.7±10.4	34.2±14.6	0.096
Onset age(years)	19.5±10.6	27.1±14.5	0.102
eGFR (ml/min/1.73m^2^)	118.6±23.9	118.7±19.8	0.994
SBP(mmHg)	110.2±11.1	109.1±12.6	0.809
QTc (sec)	0.452±0.057	0.431±0.034	0.256
BMI(kg/m2)	21.01±4.55	22.12±3.43	0.491
Symptom (n, %)			
fatigue	6, 40.0	12, 66.7	0.170
dizziness	2, 13.3	4, 22.2	0.665
fainting	0, 0	4, 22.2	0.108
muscle weakness	13, 86.7	13, 78.8	0.413
cramps	2,13.3	1,5.6	0.579
muscle stiffness/pain	7,46.7	5,27.8	0.300
arthralgia	2, 13.3	1, 5.6	0.579
nocturia	4, 26.7	12, 66.7	0.037
polyuria	3, 20.0	4, 22.2	1.000
thirst	5, 35.7	7, 38.9	1.000
dyspnea	0, 0	5, 27.8	0.049
Serum(mmol/L)			
Potassium	3.20±0.61	3.11±0.61	0.669
Natrium	138.3±1.9	138.7±2.1	0.520
Chloride	97.1±4.0	96.1±3.0	0.411
Magnesium	0.721±0.162	0.680±0.191	0.513
Calcium	2.486±0.104	2.418±0.128	0.108
Phosphate	1.29±0.27	1.17±0.34	0.279
Hypomagnesemia (n,%)	8, 53.3	12, 66.7	0.493
Blood gas			
pH	7.461±0.041	7.473±0.037	0.386
cHCO_3_^-^(mmol/L)	27.7±3.5	30.1±4.3	0.092
ABE(mmol/L)	3.64±2.40	5.85±3.15	0.033
Plasma PGEM (pg/ml)	8.405(3.820, 11.958)	7.803(3.708, 18.678)	0.830
HCT			
ΔClearance Cl(ml/min)	0.59(0.28, 1.18)	0.10(-0.03, 0.45)	0.021
24h Urine(mmol/d)			
Potassium	179.0±55.7	272.8±89.8	0.002
Natrium	92.7±50.1	116.7±94.8	0.387
Chloride	211.2±39.8	304.1±83.6	<0.001
magnesium	5.16±2.16	6.02±5.05	0.558
Calcium	1.68(0.52, 2.76)	1.05(0.47, 1.99)	0.222
Phosphate	18.12(11.00, 29.52)	14.91(10.12, 22.44)	0.423
Ca/Cr ratio	0.053(0.025, 0.111)	0.062(0.035, 0.115)	0.596
Renin–Angiotensin-Aldosterone System			
Upright renin(ng/ml/h)	2.47(1.13, 12.00)	3.07(1.35, 4.44)	0.509
Angiotensin II (pg/ml)	291.62(128.82, 382.20)	222.01(155.18, 375.95)	0.895
Aldosterone (ng/dl)	16.89(13.45, 25.04	19.53(16.10, 28.36)	0.227

Abbreviations: ABE, actual base excess; eGFR, estimated glomerular filtration rate, ml/min/1.73m2; SBP, systolic blood pressure; PGEM, prostaglandin E2 metabolite; ΔClearance Cl, the absolute increases in the chloride learance rate; HCT, hydrochlorothiazide. The higher PGEM group: Urinary PGEM level higher than 0.55 ng/mL for male and 0.16 ng/mL for female; The lower PGEM group: Urinary PGEM level≤0.55ng/mL for male and ≤0.16 ng/mL for female. Normal range of Upright renin: 0.93–6.56ng/ml/h; Angiotensin II: 25.3–143.3pg/ml; Aldosterone: 6.5–29.6ng/dl.

The physical function of NCC was measured using the thiazide test. After treatment with HCT, chloride clearance disorders were more severe in patients with higher PGEM levels than in those with lower PGEM levels (median 0.10, P_25_-P_75_: -0.03~0.45 vs. Median 0.59, P_25_-P_75_: 0.28~1.18, union: mL/min; *P* = 0.021).

The elevation of plasma AngII (71.8%) and PRA (23.5%) levels was observed in patients with GS. The logistic regression model showed that neither urinary nor plasma PGEM were associated with RAAS levels.

## Discussion

GS has traditionally been thought to be a relatively benign disorder that could be treated by potassium and magnesium supplementation. Since the first report of normal PGE2 levels in patients diagnosed with GS by clinical manifestations in 1995[3], few studies have monitored the PGE2 levels associated with GS. Although no positive recommendation has been advocated by physicians, COX2 inhibitors, which are used to block PGE2 production, were reported to be helpful for GS patients[4, 5]. To the best of our knowledge, this is the first direct evidence of elevated PGEM levels in patients with genetically confirmed GS. After adjusting for the gender difference, urinary PGEM was correlated with plasma chloride and magnesium levels and urinary calcium excretion. The most interesting finding was that higher urinary PGEM levels indicated more severe clinical manifestations and NCC dysfunction.

We first demonstrated higher PGEM levels in GS patients relative to healthy controls of the same gender. Although Luthy et al.[3] described normal PGE-2 levels in 11 GS patients diagnosed by clinical manifestations, the diagnosis was not confirmed by DNA sequencing and the sample size was limited. Furthermore, among these patients, one male patient showed excessively higher levels of PGE2 than his sister. We also enrolled two siblings from one family with the same gene mutation (D486N). The urinary PGE2 and PGEM levels of the brother were more than 7 times that of his sister, accompanied by more severe clinical manifestations. In our study, significantly higher levels of PGE2 and PGEM were observed not only in male patients but also in male healthy volunteers. Therefore, the limited sample size and overlooked gender differences might have masked the high PGE2 levels in GS patients in the study by Luthy et al. In fact, a mild elevation of urinary PGE2 levels in GS patients was reviewed by Jeck et al. in 2005[19]. Indirect evidence of the benefits of indomethacin treatment in GS patients was demonstrated in several case series[5, 12, 20, 21], but a few series measured PGE2 levels[4, 19, 22, 23]. However, the results of urinary PGE2 excretion are controversial; Larkins et al. found increased urinary prostaglandin (PGE2) excretion in two children who showed a good response to indomethacin (2059 ng/24 h/1.73 m^2^ in Case 1 and 3609 ng/24 h/1.73 m^2^ in Case 2; the normal range is 48–394 ng/24 h/1.73 m^2^)[4]. Peters et al. reported normal PGE2 excretion (21±12, normal range 4–27 ng/h/1.73 m^2^) but higher PGEM excretion (944±656, normal range 62–482 ng/h/1.73 m^2^)[23].

The mechanism of elevated PGE2 levels in GS patients is not clear. Existing evidence indicates that increased PGE2 might be secondary to salt loss rather than wholly due to the primary disease. Even in BS, a traditional hyperprostaglandin E syndrome, not all patients show enhanced synthesis or excretion of PGE[24]. However, in the early 1970s, Nivet et al.[25] noticed that hyperprostaglandinuria was not specific to BS, sodium depletion and water deprivation were found in individuals without BS. In 2014, Larkins et al. reported two female GS patients who exhibited metabolic alkalosis and hypokalemia at 5 months of age with high urine PGE2 levels. PGE2 excretion significantly decreased after a bolus of normal saline (20 mL/kg) and following 48 h of intravenous fluids[4]. Animal studies have also confirmed enhanced renal COX2 and PGE2 protein and mRNA expression in a salt-losing tubulopathy model[14, 26, 27]. Inactivating mutations of NCC in patients with GS were similar to the chronic administration of thiazide diuretics, which specifically block NCC in the DCT. In male SD rats, the diuretic HCT increased prostanoid excretion and could be blunted by the COX2 inhibitor rofecoxib[28].

The association of urinary PGEM with electrolyte disorders and NCC dysfunction can be explained by the regulation of mechanisms underlying COX2 synthesis. First, under acute salt/volume depletion, COX2 expression is significantly up-regulated in macula densa cells through a mechanism by which low intracellular chloride levels induce COX2 via p38 MAPK[22]. Second, chronic mild decreases of effective circulatory volume could increase plasma vasopressin to stimulate prostaglandin synthesis[29, 30]. Third, a high urinary flow rate, such as in polyuria, leads to an increase in fluid shear stress (FSS), which mediates the release of PGE2 by the cortical collecting duct (CCD) to enhance renal sodium excretion. Tubular flow biomechanically regulates PGE2 release and COX-2 expression through the stimulation of neutral-sphingomyelinase (N-SM) activity[31]. Additionally, salt loss induces the enhanced expression of the prorenin receptor (PRR), and AngII also activates the expression and activity of COX2[32]. Through EP_1_ and EP_2_ receptors[33], PGE2 can inhibit sodium reabsorption in the collecting duct; therefore, the inhibition of PGE2 directly regulates epithelial sodium channels and increases renal sodium absorption[34].

This was the first study to establish the relationship between PGEM and NCC function in humans. A higher urinary PGEM level can be an indicator of severe disease. It could explain the high levels of PGE2 once treatment with indomethacin reduces PGE2 excretion to subnormal levels and the partial reversal of the clinical syndrome[35]. In this study, the thiazide test was used to evaluate the in vivo function of the NCC. A blunt reaction to HCT indicated impaired reabsorption function of the DCT. The relationship between the NCC disorder and the clinical manifestations of GS was shown in our previous studies [15, 17, 18]. Because of the limited sample size, we didn’t find significant association between genotype and phenotype, nor the relationship between PGE2 levels and the mutations (including the type of mutations: homozygous, compound heterozygous and heterozygous; locations of NCC protein encoding by different mutations: cytoplasmic sequence, transmembrane regions, intracellular or extracellular). It would be valuable to evaluate the relationship between gene mutations and PGE2 levels in more patients with GS. Indomethacin has been reported to markedly reduce plasma renin concentrations but does not change the plasma aldosterone concentration[12]. We also failed to determine the correlation between circulating RAAS activity and PGE2. Because the RAAS is regulated by multiple factors[36], a more precise study should be performed to establish their relationship.

As a stable surrogate marker of PGE2 levels, urinary PGEM has been used as a biological marker of pancreatic cancer[37] and ulcerative colitis[27]. Urine PGEM could be a potential therapeutic target in GS patients. Both selective and nonselective inhibitors of COX2 can improve refractory hypokalemia and decrease plasma RAAS activity[12], but they also increase the risk of eGFR decreases. In the randomized crossover study conducted by Blanchard et al., even a low dose of 75 mg indomethacin for only 6 weeks decreased the eGFR by 10.0 mL/min/1.73 m^2^, which is slightly greater than that observed with amiloride[12]. However, PGE2 levels in urine and plasma were never measured in these patients. According to our study, the PGEM level varied between patients, and we suggest a COX2 inhibitor as a more appropriate choice for patients with elevated PGEM levels. For long-term treatment, the gastrointestinal tolerability and cardiovascular effects should also be taken into consideration.

### Limitations

There are some limitations in this study. (1) Many patients had received potassium and magnesium supplementation before admission, which may have improved their electrolyte disturbance and affected the levels of PGE2 and PGEM. (2) PGE2 and PGEM levels varied in different GS patients and controls as reported by different research groups. Based on the detection methods, data from different reports were not comparable. For a stable and reliable reference range and cut-off value of urinary PGEM, a much larger sample size and diverse population are needed. (3) The effect of COX2 inhibitors on circulating PGE2 levels was unclear. To strongly support the use of indomethacin or other COX inhibitors, a well-designed clinical trial including the monitoring of PGE2 levels as well as renal PGE2 synthesis and plasma renin levels is needed.

## Conclusions

Higher urinary PGE2 metabolite levels indicated more severe clinical manifestations and NCC dysfunction in GS patients. COX2 inhibition might be a potential therapeutic target in GS patients with elevated PGEM levels.

## Supporting information

S1 Table *SLC12A3* mutations identified in 39 Chinese patients with Gitelman syndrome. Abbreviations and note: M: male; F: female; del: deletion; ins: insertion; CoHet: compound heterozygous; Het, heterozygous; Homo: homozygous. GenBank accession number NM_000339.2 is used as a reference sequence. ^*^Reported in Jiang L, et al. Endocr Pract, 2015, 21(9): 1017–25. ^**#**^Reported in Yuan T, et al. Endocr Connect, 2017. DOI: 10.1530/ec-17-0014. ^**$**^Reported in Jiang L, et al. Am J Nephrol, 2014, 39(4): 357–366.
(DOC)

S2 Table Urinary and plasma PGE2 (PGEM) levels of individual subjects. Abbreviations: M: male; F: female; HC: healthy controls; GS: Gitelman syndrome; PGE2: prostaglandin E2; PGEM: prostaglandin E2 metabolite.
(DOC)
