# Supplementary material for: Increased urinary prostaglandin E2 metabolite: A potential therapeutic target of Gitelman syndrome
Source: PLoS One. 2017 Jul 10;12(7):e0180811. doi: 10.1371/journal.pone.0180811 (PMC5507263; doi:10.1371/journal.pone.0180811)
Supplement: S1 Table — Abbreviations and note: M: male; F: female; del: deletion; ins: insertion; CoHet: compound heterozygous; Het, heterozygous; Homo: homozygous. GenBank accession number NM_000339.2 is used as a reference sequence. *Reported in Jiang L, et al. Endocr Pract, 2015, 21(9): 1017–25. #Reported in Yuan T, et al. Endocr Connect, 2017. DOI: 10.1530/ec-17-0014. $Reported in Jiang L, et al. Am J Nephrol, 2014, 39(4): 357–366. (DOC) [file pone.0180811.s001.doc]

**S1 Table. *SLC12A3* mutations identified in 39 Chinese patients with Gitelman syndrome**

| **Patient NO.** | **Gender** | **age (y)** | **Position** | **Nucleotide change** | **Homo/Het/CoHet** | **Predicted protein change** | **Reference** |
| --- | --- | --- | --- | --- | --- | --- | --- |
| Homozygous（n=5） | | | | | | | |
| 3* | F | 17 | Exon 12 | c.1456G>A | Homo | Asp486Asn | Simon DB. Nat Genet. 1996;12(1):24-30. |
| 10* | F | 36 | Exon 1 | c.179C>T | Homo | Thr60Met | Maki N. Nephrol Dial Transplant. 2004;19(7):1761-6. |
| 11* | M | 38 | Exon 1 | c.179C>T | Homo | Thr60Met | Maki N. Nephrol Dial Transplant. 2004;19(7):1761-6. |
| 31^$^ | M | 25 | Exon 1 | c.179C>T | Homo | Thr60Met | Maki N. Nephrol Dial Transplant. 2004;19(7):1761-6. |
|  |  |  | Exon 15 | c.1964 G>A | Homo | Arg655His | Simon DB. Nat Genet. 1996;12(1):24-30. |
| 5* | F | 26 | Exon 1 | c.48 C>A | Homo | Cys16X | Jiang L. Endocr Pract, 2015, 21(9): 1017-25. |
|  |  |  | Exon 5 | c.602G>A | Het | Gly201Asp | Jiang L. Endocr Pract, 2015, 21(9): 1017-25. |
|  |  |  | Exon 2 | c.391G>A | Het | Glu131Lys | Jiang L. Endocr Pract, 2015, 21(9): 1017-25. |
| Compound Heterozygous（n=22） | | | | | | | |
| 1* | M | 12 | Exon 5 | c.644T>C | Cohet | Leu215Pro | Lemmink. Kidney Int. 1998;54(3):720-30. |
|  |  |  | Exon 21 | c.2532G>A |  | Trp844X | Lin SH. J Clin Endocrinol Metab. 2005;90(5):2500-7. |
| 7* | M | 19 | Exon 10 | c.1315 G>A | CoHet | Gly439Ser | Mastroianni N. Am J Hum Genet. 1996;59(5):1019-26. |
|  |  |  | Exon 15 | c.1844 C>T |  | Ser615Leu | Cruz DN. Kidney Int. 2001;59(2):710-7. |
| 8* | M | 25 | Exon 3 | c.452 G>A | CoHet | Trp151X | Jiang L. Endocr Pract, 2015, 21(9): 1017-25. |
|  |  |  | Exon 20 | c.2398 G>A |  | Gly800Arg | Jiang L. Endocr Pract, 2015, 21(9): 1017-25. |
|  |  |  | Exon 9 | c.1108 G>C |  | Ala370Pro | Jiang L. Endocr Pract, 2015, 21(9): 1017-25. |
| 9* | M | 14 | Exon 21 | c. 2454_2461delCAAGGCCC | CoHet | 819frameshift | Jiang L. Endocr Pract, 2015, 21(9): 1017-25. |
|  |  |  | Exon 23 | c.2738G>A |  | Arg913Gln | Tseng M H. J Clin Endocrinol Metab, 2012, 97(8): E1478-82. |
| 12^#^ | M | 27 | Exon 3 | c.486-490delTACGGinsA | CoHet | 162frameshift | Qin L. Nephrology (Carlton). 2009;14(1):52-8. |
|  |  |  | Exon 10 | c.1288 T>G |  | Cys430Gly | Shao L. Nephron Physiol. 2008;108(3):29-36. |
|  |  |  | Exon 16 | c.1975G>A |  | Val659Met | Yuan T, et al. Endocr Connect, 2017. DOI: 10.1530/ec-17-0014. |
| 13* | M | 44 | Exon 10 | c.1283 C>T | CoHet | Thr428Ile | Jiang L. Endocr Pract, 2015, 21(9): 1017-25. |
|  |  |  | Exon 12 | c.1456G>A |  | Asp486Asn | Simon. Nat Genet. 1996;12(1):24-30. |
| 14* | M | 14 | Exon 24 | c.2877_2878delAG | CoHet | Arg959fs | Lin. Am J Kidney Dis, 2004, 43(2): 304-12. |
|  |  |  | Exon 4 | c.587G>T |  | Gly196Val | Shao L. Nephron Physiol. 2008;108(3):29-36. |
| 15 | M | 17 | Exon 1 | c.179C>T | CoHet | p.Thr60Met | Maki N. Nephrol Dial Transplant. 2004;19(7):1761-6. |
|  |  |  | Exon13 | c.1602 A>C |  | Asn534Lys | This study |
| 16 | M | 37 | Exon 1 | c.248G>A | CoHet | Arg83Gln | Vargas-Poussou R. J Am Soc Nephrol. 2011;22(4):693-703. |
|  |  |  | Exon 24 | c.2782C>T |  | Arg928Cys | Lemmink. Kidney Int. 1998;54(3):720-30. |
| 19 | M | 15 | Exon 12 | c.1456G>A | CoHet | Asp486Asn | Simon DB. Nat Genet. 1996;12(1):24-30. |
|  |  |  | Exon 6 | c.806 ins TTGGCGTGGTCTCGGTCA |  | 269 ins IGVVSV | Tajima T. Endocr J. 2002;49(1):91-6. |
| 22* | M | 40 | Exon 12 | c.1456G>A | CoHet | Asp486Asn | Simon DB. Nat Genet. 1996;12(1):24-30. |
|  |  |  | Exon 10 | c.1195 C＞T |  | Arg399Cys | Cruz DN,. Kidney Int. 2001;59(2):710-7. |
| 24* | M | 46 | Exon 25 | c.2927 C>T | CoHet | Ser976Phe | Jang HR. Kidney Int. 2006;70(4):813-7. |
|  |  |  | Exon 16 | c.2029G>A |  | Val677Met | Syren ML. Hum Mutat. 2002;20(1):78. |
| 25* | M | 18 | Intron3 | c.506-1G>A | CoHet | splice defect | Abuladze N. J Am Soc Nephrol. 1998;9(5):819-26. |
|  |  |  | Exon 3 | c.496G>A |  | Leu170Gln | Jiang L. Endocr Pract, 2015, 21(9): 1017-25. |
| 26* | M | 16 | Exon 1 | c.209A>G | CoHet | Tyr70Cys | Jiang L. Endocr Pract, 2015, 21(9): 1017-25. |
|  |  |  | Exon 22 | c.2581C>T |  | Arg861Cys | Lemmink. Kidney Int. 1998;54(3):720-30. |
| 27* | M | 23 | Exon 17 | c.2098C>G | CoHet | Leu700Val | Jiang L. Endocr Pract, 2015, 21(9): 1017-25. |
|  |  |  | Exon 23 | c.2738G>A |  | Arg913Gln | Tseng M H. J Clin Endocrinol Metab, 2012, 97(8): E1478-82. |
| 29 | F | 30 | Exon 16 | c.1946 C>T | CoHet | Thr649Met | Ji W. Nat Genet. 2008;40(5):592-9. |
|  |  |  | Exon 15 | c.1909 C>T |  | His637Tyr | This study |
| 30 | F | 53 | Exon 24 | c.2782C>T | CoHet | Arg928Cys | Lemmink. Kidney Int. 1998;54(3):720-30. |
|  |  |  | Exon 15 | c.1924C>T |  | Arg642Cys | Cruz DN. Kidney Int. 2001;59(2):710-7. |
| 33 | M | 49 | Exon 8 | c.1077 C>G | CoHet | Asn359Lys | Qin L. Nephrology (Carlton). 2009;14(1):52-8. |
|  |  |  | Exon 15 | c.1850 A>G |  | Gln617Arg | This study |
| 35 | F | 20 | Exon 10 | c.1315 G>A | CoHet | Gly439Ser | Mastroianni N. Am J Hum Genet. 1996;59(5):1019-26. |
|  |  |  | Exon 15 | c.1924C>T |  | Arg642Cys | Cruz DN. Kidney Int. 2001;59(2):710-7. |
| 36 | F | 42 | Exon 22 | c.2582 G>A | CoHet | Arg861His | Aoi N. Endocrine. 2007;31(2):149-53. |
|  |  |  | Exon 14 | c.1698 C>A |  | Asn566Lys | Jiang L. Am J Nephrol, 2014, 39(4): 357-366. |
| 37 | F | 60 | Exon 4 | c.539C>A | CoHet | Thr180Lys | Monkawa T. J Am Soc Nephrol. 2000;11(1):65-70. |
|  |  |  | Exon 1 | c.179C>T |  | Thr60Met | Maki N. Nephrol Dial Transplant. 2004;19(7):1761-6. |
| 38 | M | 23 | Exon 6 | c.815T>C | CoHet | Leu272Pro | Ji W. Nat Genet. 2008;40(5):592-9. |
|  |  |  | Intron7/ Exon 8 | c.965-1_976delinsACCGAAAATTTT |  | splice defect | Qin L. Nephrology (Carlton). 2009;14(1):52-8 |
| Heterozygous（n=12） | | | | | | | |
| 23* | M | 48 | Exon 15 | c.1844 C>T | Het | Ser615Leu | Cruz DN. Kidney Int. 2001;59(2):710-7. |
| 17 | F | 23 | Exon 12 | c.1456G>A | Het | Asp486Asn | Simon DB. Nat Genet. 1996;12(1):24-30. |
| 18^$^ | F | 21 | Exon 14 | c.1698 C>A | Het | Asn566Lys | Jiang L. Am J Nephrol, 2014, 39(4): 357-366. |
| 2* | M | 73 | Exon 12 | c.1456G>A | Het | Asp486Asn | Simon DB. Nat Genet. 1996;12(1):24-30. |
| 4 | M | 24 | Exon 16 | c1964G>T | Het | Arg655Leu | Simon DB. Nat Genet. 1996;12(1):24-30. |
| 6 | F | 32 | Exon 23 | c.2738G>A | Het | Arg913Gln | Tseng M H. J Clin Endocrinol Metab, 2012, 97(8): E1478-82. |
| 20^$^ | M | 28 | Exon 23 | c.2738G>A | Het | Arg913Gln | Tseng M H. J Clin Endocrinol Metab, 2012, 97(8): E1478-82. |
| 21 | F | 42 | Exon 24 | c.2782C>T | Het | Arg928Cys | Lemmink. Kidney Int. 1998;54(3):720-30. |
| 28 | M | 15 | Exon 12 | c.1456G>A | Het | Asp486Asn | Simon DB. Nat Genet. 1996;12(1):24-30. |
| 39 | F | 31 | Exon 24 | c.2782C>T | Het | Arg928Cys | Lemmink. Kidney Int. 1998;54(3):720-30. |
| 34 | M | 24 | Exon 12 | c.1456G>A | Het | Asp486Asn | Simon DB. Nat Genet. 1996;12(1):24-30. |
| 32 | M | 52 | Exon 6 | c.806 ins TTGGCGTGGTCTCGGTCA | Het | 269 ins IGVVSV | Tajima T. Endocr J. 2002;49(1):91-6. |

Abbreviations and note: M: male; F: female; del: deletion; ins: insertion; CoHet: compound heterozygous; Het, heterozygous; Homo: homozygous.

GenBank accession number NM_000339.2 is used as a reference sequence.

*Reported in Jiang L, et al. Endocr Pract, 2015, 21(9): 1017-25.

#Reported in Yuan T, et al. Endocr Connect, 2017. DOI: 10.1530/ec-17-0014.

$Reported in Jiang L, et al. Am J Nephrol, 2014, 39(4): 357-366.
