# Supplementary material for: Increased urinary prostaglandin E2 metabolite: A potential therapeutic target of Gitelman syndrome
Source: PLoS One. 2017 Jul 10;12(7):e0180811. doi: 10.1371/journal.pone.0180811 (PMC5507263; doi:10.1371/journal.pone.0180811)
Supplement: S2 Table — Abbreviations: M: male; F: female; HC: healthy controls; GS: Gitelman syndrome; PGE2: prostaglandin E2; PGEM: prostaglandin E2 metabolite. (DOC) [file pone.0180811.s002.doc]

**S2 Table. Urinary and plasma PGE2 (PGEM) levels of individual subjects.**

| **Patient NO.** | **Group** | **gender** | **Age**  **(y)** | **Urinary PGE2 (ng/mL)** | **Urinary PGEM (ng/mL)** | **Plasma PGEM (pg/mL)** | **Serum K^+^ (mol/L)** | **Serum Mg^2+^ ( mol/L)** | **24h Urine K^+^ (mmol/d)** |
| --- | --- | --- | --- | --- | --- | --- | --- | --- | --- |
| NC1 | HC | M | 26 | 0.6719 | 0.6917 | 8.5000 | 4.0 | 0.85 | 57.10 |
| NC2 | HC | M | 32 | 1.2292 | 0.9376 | 4.4830 | 4.1 | 0.93 | 59.40 |
| NC3 | HC | F | 26 | 0.3283 | 0.2456 | 2.5805 | 3.4 | 0.78 | 28.10 |
| NC4 | HC | F | 25 | 0.2061 | 0.2629 | 2.3125 | 4.4 | 0.79 | 32.10 |
| NC5 | HC | M | 29 | 2.1362 | 0.3239 | 11.3650 | 4.3 | 0.91 | 32.40 |
| NC6 | HC | M | 26 | 0.3748 | 0.2470 | 9.0135 | 3.9 | 0.89 | 31.50 |
| NC7 | HC | F | 26 | 0.1500 | 0.1543 | 4.6250 | 4.3 | 0.78 | 30.40 |
| NC8 | HC | M | 28 | 12.6726 | 0.4067 | 3.2730 | 4.2 | 0.88 | 46.10 |
| NC9 | HC | M | 32 | 0.0956 | 0.1608 | 2.6270 | 4.5 | 1.00 | 30.00 |
| NC10 | HC | F | 26 | 0.0976 | 0.0239 | 2.5680 | 4.8 | 0.81 | 40.90 |
| NC11 | HC | F | 31 | 0.0918 | 0.0245 | 1.5650 | 3.9 | 0.76 | 36.72 |
| NC12 | HC | F | 26 | 0.1461 | 0.1526 | 1.1930 | 4.3 | 0.89 | 40.90 |
| NC13 | HC | F | 26 | 0.2118 | 0.1090 | 0.8170 | 4.0 | 0.86 | 22.80 |
| NC14 | HC | F | 26 | 0.0683 | 0.0252 | 0.6035 | 4.1 | 0.88 | 20.20 |
| NC15 | HC | M | 29 | 0.3146 | 0.3190 | 0.4875 | 4.5 | 0.86 | 39.40 |
| NC16 | HC | M | 26 | 0.1824 | 0.1091 | 0.4355 | 4.1 | 0.84 | 45.30 |
| NC17 | HC | F | 27 | 0.1076 | 0.0871 | 0.3710 | 4.3 | 0.81 | 29.90 |
| NC18 | HC | F | 26 | 0.0873 | 0.0274 | 0.1270 | 3.8 | 0.81 | 27.60 |
| NC19 | HC | F | 26 | 0.4217 | 0.1122 | 0.1085 | 4.0 | 0.82 | 33.80 |
| NC20 | HC | M | 27 | 0.1802 | 0.0468 | 0.0165 | 4.0 | 0.86 | 23.90 |
| 1 | GS | M | 12 |  |  | 9.6560 | 2.8 | 0.75 | 72.36 |
| 2 | GS | M | 73 |  |  | 2.2905 | 3.3 | 0.45 | 85.96 |
| 3 | GS | F | 17 |  |  | 6.1400 | 3.0 | 0.58 | 138.24 |
| 4 | GS | M | 24 |  |  | 4.0920 | 2.7 | 0.68 | 57.20 |
| 5 | GS | F | 26 |  |  | 1.0690 | 3.1 | 0.58 | 67.70 |
| 6 | GS | F | 32 |  |  | 5.3270 | 4.2 | 0.83 | 65.83 |
| 7 | GS | M | 19 | 0.4289 | 0.2715 |  | 3.5 | 0.69 | 144.63 |
| 8 | GS | M | 25 | 0.4038 | 0.4200 | 2.1985 | 2.9 | 0.56 | 111.93 |
| 9 | GS | M | 14 | 0.7655 | 0.5274 | 80.7275 | 3.6 | 0.72 | 91.39 |
| 10 | GS | F | 36 | 0.1696 | 0.0966 | 14.1250 | 3.5 | 0.88 | 100.40 |
| 11 | GS | M | 38 | 0.1806 | 0.1915 | 9.3960 | 2.1 | 0.91 | 59.64 |
| 12 | GS | M | 27 | 0.4882 | 0.3467 |  | 2.5 | 0.65 | 93.93 |
| 13 | GS | M | 44 | 1.0029 | 0.4859 | 10.4310 | 3.8 | 0.84 | 94.22 |
| 14 | GS | M | 14 | 0.1242 | 0.0574 | 8.4035 | 2.3 | 0.73 | 146.16 |
| 15 | GS | M | 17 | 1.4542 | 0.4996 | 12.3040 | 2.9 | 0.76 | 52.64 |
| 16 | GS | M | 37 | 0.1514 | 0.3983 | 11.6125 | 3.4 | 0.43 | 222.00 |
| 17 | GS | F | 23 | 0.1512 | 0.1235 | 8.4045 | 2.8 | 0.49 | 40.50 |
| 18 | GS | F | 21 | 0.1271 | 0.1179 | 7.5235 | 4.1 | 0.84 | 95.37 |
| 19 | GS | M | 15 | 0.2904 | 0.4378 | 5.4410 | 3.0 | 0.53 | 62.20 |
| 20 | GS | M | 28 | 0.1144 | 0.1097 | 1.5055 | 3.8 | 0.97 | 35.48 |
| 21 | GS | F | 42 | 0.0723 | 0.0682 | 0.3375 | 3.8 | 0.82 | 39.52 |
| 22 | GS | M | 40 | 0.5046 | 0.8213 | 39.5890 | 4.4 | 0.47 | 54.00 |
| 23 | GS | M | 48 | 0.9603 | 1.5770 | 4.1845 | 3.4 | 0.46 | 97.65 |
| 24 | GS | M | 46 | 0.8035 | 0.8139 | 18.1020 | 2.9 | 0.76 | 82.42 |
| 25 | GS | M | 18 | 1.4073 | 1.8923 | 17.3805 | 2.8 | 0.58 | 90.72 |
| 26 | GS | M | 16 | 42.0180 | 1.2412 | 13.1860 | 2.4 | 0.71 | 46.95 |
| 27 | GS | M | 23 | 8.6323 | 1.0927 | 8.1245 | 3.2 | 0.88 | 120.80 |
| 28 | GS | M | 15 | 4.8099 | 0.5774 |  | 3.0 | 0.59 | 66.00 |
| 29 | GS | F | 30 | 0.2082 | 0.1603 |  | 2.8 | 0.98 | 164.90 |
| 30 | GS | F | 53 | 0.2745 | 1.5582 | 44.5760 | 4.1 | 0.63 | 455.60 |
| 31 | GS | M | 25 | 0.3894 | 0.8683 | 20.4040 | 2.1 | 1.05 | 136.80 |
| 32 | GS | M | 52 | 0.3318 | 0.6126 | 7.4820 | 2.9 | 0.53 | 75.66 |
| 33 | GS | M | 49 | 0.2966 | 0.5566 | 6.9655 | 2.9 | 0.33 | 46.92 |
| 34 | GS | M | 24 | 2.0605 | 2.7443 | 5.0495 | 2.7 | 0.75 | 134.40 |
| 35 | GS | F | 20 | 0.6175 | 0.6796 | 2.2765 | 2.9 | 0.69 | 103.38 |
| 36 | GS | F | 42 | 0.2448 | 0.1780 | 2.0795 | 3.9 | 0.84 | 156.60 |
| 37 | GS | F | 60 | 0.2890 | 0.3499 | 1.3835 | 3.8 | 0.81 | 94.52 |
| 38 | GS | M | 23 | 16.2294 | 3.0468 |  | 2.5 | 0.48 | 56.70 |
| 39 | GS | F | 31 | 1.0186 | 0.5595 |  | 3.2 | 0.70 |  |

Abbreviations: M: male; F: female; HC: healthy controls; GS: Gitelman syndrome; PGE2: prostaglandin E2; PGEM: prostaglandin E2 metabolite.
